# A self-training interpretable cell type annotation framework using specific marker gene

**DOI:** 10.1093/bioinformatics/btae569

**Published:** 2024-09-23

**Authors:** Hegang Chen, Yuyin Lu, Yanghui Rao

**Affiliations:** School of Computer Science and Engineering, Sun Yat-sen University, Guangzhou 510006, China; School of Computer Science and Engineering, Sun Yat-sen University, Guangzhou 510006, China; School of Computer Science and Engineering, Sun Yat-sen University, Guangzhou 510006, China

## Abstract

**Motivation:**

Recent advances in sequencing technology provide opportunities to study biological processes at a higher resolution. Cell type annotation is an important step in scRNA-seq analysis, which often relies on established marker genes. However, most of the previous methods divide the identification of cell types into two stages, clustering and assignment, whose performances are susceptible to the clustering algorithm, and the marker information cannot effectively guide the clustering process. Furthermore, their linear heuristic-based cell assignment process is often insufficient to capture potential dependencies between cells and types.

**Results:**

Here, we present *I*nterpretable *C*ell *T*ype *A*nnotation based on *s*elf-training (sICTA), a marker-based cell type annotation method that combines the self-training strategy with pseudo-labeling and the nonlinear association capturing capability of Transformer. In addition, we incorporate biological priori knowledge of genes and pathways into the classifier through an attention mechanism to enhance the transparency of the model. A benchmark analysis on 11 publicly available single-cell datasets demonstrates the superiority of sICTA compared to state-of-the-art methods. The robustness of our method is further validated by evaluating the prediction accuracy of the model on different cell types for each single-cell data. Moreover, ablation studies show that self-training and the ability to capture potential dependencies between cells and cell types, both of which are mutually reinforcing, work together to improve model performance. Finally, we apply sICTA to the pancreatic dataset, exemplifying the interpretable attention matrix captured by sICTA.

**Availability and implementation:**

The source code of sICTA is available in public at https://github.com/nbnbhwyy/sICTA. The processed datasets can be found at https://drive.google.com/drive/folders/1jbqSxacL_IDIZ4uPjq220C9Kv024m9eL. The final version of the model will be permanently available at https://doi.org/10.5281/zenodo.13474010

## 1 Introduction

Identifying different cell types in complex tissues contributes to a deeper understanding of the role of cells in a variety of biological processes (Fawkner-Corbett *et al.* 2021, Liu *et al.* 2021). Traditionally, cell sorting and microscopy have been used to isolate cell types, followed by molecular analyses of the sorted cells using methods such as mRNA or protein measurements (Lubeck and Cai 2012, Maestre-Batlle *et al.* 2017). Over the years, the biological field has accumulated a large number of cell-specific features, including marker genes, which can be used to distinguish between various cell types in new tissue (Franzén *et al.* 2019, Zhang *et al.* 2019a). Recent advances in single-cell RNA sequencing (scRNA-seq) have allowed us to monitor biological systems at a much higher resolution. Nevertheless, the process of single-cell type annotation and analysis manually is both cumbersome and inefficient.

Recently, an increasing number of automated pipelines have been developed to facilitate cell type annotation, mainly divided into marker-based and reference-based approaches. Specifically, the key idea of reference-based approaches is to train a classification model using reference datasets and then migrate it to a new dataset, such as scmap (Kiselev *et al.* 2018), SingleR (Aran *et al.* 2019), scPred (Alquicira-Hernandez *et al.* 2019), scDeepInsight (Jia *et al.* 2023), and CIForm (Xu *et al.* 2023). However, such approaches require that the reference and target datasets resemble each other, which often pose a problem in scRNA-seq studies (Ianevski *et al.* 2022, Jia *et al.* 2023). In addition, scSHAPR (Lewinsohn *et al.* 2023) integrates multiple single-cell annotation methods based on marker genes and reference datasets, and propagates the confidence labels generated by these methods using semi-supervised graph convolutional networks. Although it is an interesting attempt to improve the model performance by integrating different types of cell annotation methods, this integration strategy requires more external information such as reference datasets and marker information of cell types during training, which greatly increases the complexity of the model and is not conducive to further scaling.

The marker-based methods use a collection of known marker genes to identify different types of cells. scCATCH (Shao *et al.* 2020) employs a clustering algorithm to identify clusters of similar cells, and then it aligns cell clusters to cell types based on the overlap between specific genes of each cell cluster and marker genes of each cell type. SCINA (Zhang *et al.* 2019b) conducts single-cell type annotation by maximizing the expression values of marker genes of candidate cell types through an expectation-maximization algorithm. To distinguish between closely related cell types, Ianevski *et al.* (2022) proposed scType, a cell type annotation model that combines negative marker genes and cell type specificity score. scType first performs unsupervised cell clustering on scRNA-seq data, and then evaluates associations between cell clusters and cell types in combination with specificity scores and negative marker genes to enable accurate cell type annotation. MarkerCount (Kim *et al.* 2022) uses a marker count strategy to assess associations between cells and types and identifies rejection thresholds for each cell type through bimodal fitting. Then MarkerCount performs clustering by principal component analysis and Gaussian mixture model, followed by correction for previous assessments. The scPipeline (Mikolajewicz *et al.* 2022) method provides a single-cell data analysis toolkit that offers modular workflows for cell annotation and user-friendly analysis reports. Although these methods have been widely used for cell type annotation, most of them rely on clustering algorithms and their performance is susceptible to the effect of clustering. Furthermore, the selection of cluster-specific genes is a complex task since marker genes may correspond to multiple cell types (Ianevski *et al.* 2022). What is more, these methods divide the cell type annotation process into two phases of clustering and assignment, where the marker information of cell types cannot effectively guide the optimization of unsupervised cell clustering, which can lead to poor performance.

We further point out that previous approaches mostly use simple heuristics to assess associations between cells and cell types. For instance, Shao *et al.* (2020) used the evidence-based scoring that assesses cell and cell type associations based on the overlap between genes specific to the cell cluster and marker genes for the cell type. Building on this, Mikolajewicz *et al.* (2022) present Miko scoring that can adapt to the size of the gene set. Ianevski *et al.* (2022) proposed a scoring strategy that combines cell type specificity score and negative marker genes. In addition, Pliner *et al.* (2019) utilized marker genes to categorize cells into user-specified cell types. Then, they selected representative cells for each cell type to train a generalized linear model as a classifier. However, these simple association assessment strategies may not accurately capture potential signals in scRNA-seq data, thus inhibiting the scalability of these models on complex datasets.

**Figure 1.**
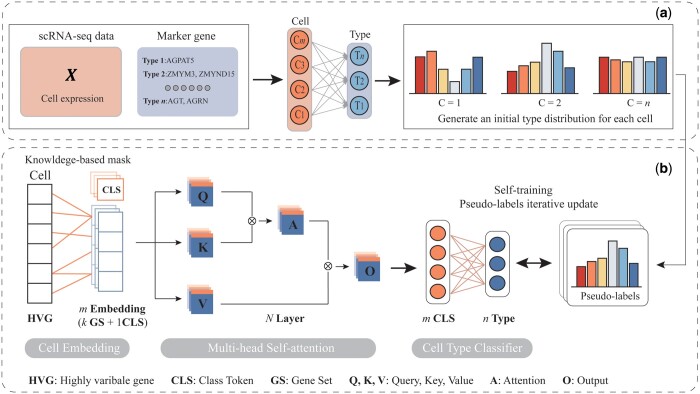
The workflow of sICTA. (a) Combining cell expression and marker gene specificity to generate pseudo-labels. (b) The downstream Transformer classifiers are first pretrained based on cell type probability distributions (pseudo-labels), followed by iterative refinement of the classifiers through a self-training framework until convergence. The sICTA takes the *a priori* knowledge from the biological domain and uses masked learnable embeddings to transform the input data (*G* genes) into *k* input tokens representing each gene set (GS) and a class token (CLS).

To overcome these challenges, we propose a new framework called *I*nterpretable *C*ell *T*ype *A*nnotation based on *s*elf-training (sICTA), which integrates the self-training strategy based on pseudo-labeling and the nonlinear association capturing capability of Transformer (Fig. 1). The pseudo-labeling-based self-training strategy first utilizes a high-quality cell-type association assessment strategy to generate pseudo-labels. Then, cell expressions and pseudo-labels are used to pretrain a downstream classifier. Finally, the classifier is iteratively optimized through a self-training process. Therefore, opposed to two-phase models that rely on clustering algorithms, our sICTA embeds the cell annotation process into a new framework that allows marker genes to better guide the optimization of model parameters. Furthermore, we use Transformer as the backbone framework for the classifier, leveraging its advanced properties in nonlinear association modeling, which enables our sICTA to better capture potential dependencies between cells and cell types. Notably, inspired by Chen *et al.* (2023), we embed biologically prior knowledge into the classifier through the attention mechanism to enhance the interpretability of the model. Extensive experiments demonstrate that our sICTA enables accurate cell type annotation and significantly outperforms state-of-the-art (SOTA) baseline models on several widely adopted benchmarks. More interestingly, we find that the attention mechanism can effectively capture the biological signals behind single-cell data even under a pseudo-labeling-based self-training framework, which provides a promising approach to elucidate the underlying biological mechanisms.

## 2 Materials and methods

### 2.1 Dataset

We perform experiments on 11 widely used scRNA-seq datasets containing different species and tissues. Eight of these data (i.e. Muraro, Baron, Stoeckius Zheng, Madissoon, Reyfman, Tirosh, and Puram) are taken from Kim *et al.* (2022), which contain manual annotations and encompass a variety of tissues and species. We remove cells labeled as unknown types from these datasets. In addition, we collect one mouse brain dataset (i.e. Zeisel) and two human blood data (i.e. Dominguez Conde and Yoshida). The marker genes for Zeisel are taken from the original author’s description (Zeisel *et al.* 2015), and the marker genes for Dominguez Conde and Yoshida are taken from the CellTypist immune cell atlas (Domínguez Conde *et al.* 2022). Supplementary Table S1 shows a detailed description of all single-cell datasets used in this work.

### 2.2 Baselines and hyperparameter settings

To better evaluate the performance of sICTA, we use five SOTA marker-based single-cell annotation methods as benchmark models, including Garnett (Pliner *et al.* 2019, https://cole-trapnell-lab.github.io/garnett), SCINA (Zhang *et al.* 2019b; https://github.com/jcao89757/SCINA), scSorter (Guo and Li 2021, https://cran.r-project.org/web/packages/scSorter/vignettes/scSorter.html), scType (Ianevski *et al.* 2022, https://github.com/IanevskiAleksandr/sc-type), and MarkerCount (Kim *et al.* 2022, https://github.com/combio-dku/MarkerCount/tree/master). scSorter is a classical marker-based single-cell annotation model inspired by the parcel sorter, which assigns individual cells to different predefined cell types based on the overexpression of marker genes. More details of method implementation are described in Supplementary Note S1.

### 2.3 Strategies for assessing the relevance between cells and cell types

In this work, pseudo-labeling generation is based on the assessment of the association between cells and cell types. Intuitively, pseudo-labeling is the training objective of the pretraining phase of sICTA, so the performance of the model benefits from high-quality pseudo-labeling. How to assess the relevance between cells and cell types based on marker genes has been extensively studied in recent years. Here, we present six commonly used association assessment strategies. Specifically, the cell expression data is a multivariate count vector $x_{i}\in R^{G}$, where *G* is the number of genes. Given *T* known cell types, each cell type $T_{j}$ has a list of marker genes, denoted as $M_{j}=\{m_{j}^{k}\},k=1..n_{j}$. Details of the adopted strategies are described below.

#### 2.3.1 Count-based relevance assessment (*Count*)

The relevance between cell $x_{i}$ and type $T_{j}$ is assessed by checking whether the marker genes in $T_{j}$ were expressed in $x_{i}$. Formally, it is defined as follows:

(1) $$R_{ij}=\sum_{k=1}^{n_{j}}1(x_{i}(m_{j}^{k})>0)$$

where $1(x_{i}(m_{j}^{k})>0)$ indicates whether the $k_{th}$ marker gene of the $j_{th}$ cell type $T_{j}$ is expressed in $x_{i}$. If $x_{i}(m_{j}^{k})>0$, then $1(x_{i}(m_{j}^{k})>0)=1$, and vice versa $1(x_{i}(m_{j}^{k})>0)=0$.

#### 2.3.2 Relevance assessment based on external knowledge and cosine similarity (*Cos*)

Here, we introduce the gene embedding E generated by previous work (Du *et al.* 2019). Based on the expression data of cells and E, the embedding representation of each cell ($c_{i}^{\mathrm{emb}}$) is obtained by weighted averaging. And the embedding representation of each cell type ($T_{j}^{\mathrm{emb}}$) is the average of its marker gene embeddings, i.e.

(2) $$c_{i}^{\mathrm{emb}}=\frac{x_{i}\cdot E}{\text{sum}(x_{i})},T_{j}^{\mathrm{emb}}=\frac{\sum_{k=1}^{n_{j}}e_{j}^{k}}{n_{j}}$$

where $e_{j}^{k}$ is the embedding of its corresponding marker gene $m_{j}^{k}$. Next, the cosine similarity between cell embeddings and type embeddings is used as the association score between them.

(3) $$R_{ij}=\text{cosine}(c_{i}^{\mathrm{emb}},T_{j}^{\mathrm{emb}})$$

#### 2.3.3 Relevance assessment based on external knowledge and logistic regression (*LR-label* and *LR-marker*)

Instead of cosine similarity, we can employ logistic regression (LR) classifiers trained on external knowledge to infer associations between cells and candidate types. In the *LR-label* strategy, we consider the cell type and its corresponding marker as a cell containing multiple genes. Therefore, we obtain the embedding representation of a cell type by averaging the marker gene embeddings and using it as a training sample to train the classifier (one training sample per class). Oppositely, in the *LR-marker* strategy, each marker gene contained in a cell type is considered to be a training sample of that cell type (multiple training samples per class).

#### 2.3.4 Pseudo-cell-based correlation assessment (*Pseudo-cell*)

This strategy is adapted from the pseudo-document generation method proposed by Meng *et al.* (2018). We map cells and genes into the same embedding space, where each cell type is modeled as a high-dimensional spherical distribution. Then pseudo-cells are generated by sampling from the type distributions. Next, pseudo-cells and their corresponding type labels are used to train downstream classifiers.

#### 2.3.5 Relevance assessment based on marker gene specificity for cell types (*Cell-type-specific*)

The cell type specificity score (S) (Ianevski *et al.* 2022) quantifies the representativeness of a marker gene for a specific cell type, i.e. a higher score indicates that the marker gene can better distinguish this cell type from others. Concretely, *S* is calculated separately for each marker gene *m*. All marker genes of candidate cell types are first pooled into a marker pool *M*, and then the cell-specific score $S_{j}$ for $M_{j}$ is calculated by the following formula:

(4) $$S_{j}=1-\frac{\left|M_{j}\right|-\min\left(\left|M\right|\right)}{\max\left(\left|M\right|\right)-\min\left(\left|M\right|\right)}$$

where $\left|M_{j}\right|$ denotes the number of cell types corresponding to the $j_{th}$ marker, while min(|M|) and max(|M|) are the minimum and maximum number of cell types corresponding to the marker genes in *M*. The expression level of each gene was then multiplied by its cell type specificity and based on this, a final cell and cell type association score $R_{ij}$ is obtained by

(5) $$\begin{array}{c} \begin{array}{c} X^{\prime }=\left({\left(Z\left(X^{T}\right)\right)}^{T}\subseteq M\right)\cdot S\\ R_{ij}=\frac{\sum_{k=1}^{n_{j}}x_{i}^{\prime }\left(m_{j}^{k}\right)}{\sqrt{n_{j}}} \end{array} \end{array}$$

where $x_{i}^{\prime }$ corresponds to a cell in $X^{\prime }$ and *Z* denotes the z-score-transformation.

### 2.4 Model pretraining

After obtaining the association scores of all cell and type pairs, we utilize them to generate pseudo-labels for the pretraining of the downstream neural network classifier. A simple strategy is to map association scores into single labels, where all cells are assigned to the cell type with the highest correlation. However, this straightforward strategy often leads to model overfitting.

To alleviate the above issue, we transformed association score between each cell and type into the probability simplex (pseudo-label) via normalization. The downstream classifier was trained by minimizing the *KL* divergence between the neural network outputs *Y* and the pseudo-labels *L* (Fig. 1a). The formula is given below:

(6) $$L=KL(L||Y)=\sum_{i}\sum_{j}l_{ij}\;\text{log}\;\frac{l_{ij}}{y_{ij}}$$

where $l_{ij}$ and $y_{ij}$ are the pseudo-label and model prediction between the $i_{th}$ and the $j_{th}$ cell types, respectively.

### 2.5 Model self-training

Due to the potential noise contained in the pseudo-labels, the pretrained classifier may not have excellent performance. The self-training step aims to address this issue. Self-training (Rosenberg *et al.* 2005) is a strategy commonly used in classical semi-supervised models, with the underlying assumption that the introduction of pseudo-labels reduces the overlap of the category probability distributions, and that it facilitates low-density separation between categories (Lee 2013).

Specifically, after the pretraining step, we further improve the performance of the classifier through a self-training process. During the self-training process, we treat the predicted probability distributions of the $t_{th}$ round as pseudo-labels of the ${\left(t+1\right)}_{th}$ round to further train the model parameters, and refine the model through multiple iterations until convergence (Fig. 1b). Formally, the loss function of the ${\left(t+1\right)}_{th}$ round of self-training is defined by

(7) $$L=KL(Y^{\left(t\right)}||Y^{\left(t+1\right)})=\sum_{i}\sum_{j}y_{ij}^{\left(t\right)}\;\text{log}\;\frac{y_{ij}^{\left(t\right)}}{y_{ij}^{\left(t+1\right)}}$$

### 2.6 Interpretable transformer

To improve the model’s ability to capture dependencies between cells and types, we select Transformer as the backbone network for downstream classifiers. Furthermore, inspired by previous work (Chen *et al.* 2023), we enhance the interpretability of cell type annotations by embedding *a priori* biological knowledge into the Transformer via the attention mechanism. Specifically, for each cell $x_{i}\in R^{G}$, we first embed it into *k* tokens, i.e.

(8) $$\begin{array}{c} \begin{array}{c} t=W^{\prime }\cdot x_{i}\\ W^{\prime }=W⊙M \end{array} \end{array}$$

where $W\in R^{k\times G}$ denotes weights of a linear transformation. To match each token to a specific path, the mask matrix $M\in {\left\{0,1\right\}}^{k\times G}$ is constrained to be a user-given gene-pathway correlation, consisting of 0 or 1. The priori gene-pathway relationships used in this work were obtained from the gene set enrichment analysis (GSEA) database (Subramanian *et al.* 2005). And ⊙ means Hadamard product. Then, we repeated the above embedding operation 100 times to increase the dimension of the embedding space. Hence, we have

(9) $$\begin{array}{c} \begin{array}{c} T=\mathrm{columnbind}\left(t_{1},t_{2},\ldots ,t_{m}\right)\\ I=\text{rowbind }(\mathrm{CLS},T) \end{array} \end{array}$$

Here, $T\in R^{k\times m}$ denotes the pathway token matrix and is spliced with the trainable class token ($\mathrm{CLS}\in R^{m}$) to generate the input matrix ($I\in R^{(1+k)\times m}$). Then, the query (Q), key (K), and value (V) matrices were obtained by linear projections of I in the multi-head self-attention layer. Subsequently, the attention matrix (A) was computed from Q and K, scaled by the inverse of the square root of the K ($d_{k}$) dimension, and activated by the softmax function, i.e.

(10) $$\begin{array}{c} \begin{array}{c} Q_{j},K_{j}=W_{j}^{q,k}\cdot I\\ Q=\text{columnbind }\left(Q_{1},\ldots ,Q_{n}\right)\\ K=\text{columnbind }\left(K_{1},\ldots ,K_{n}\right)\\ V=W^{v}\cdot I\\ A=\text{softmax}\left(\frac{Q\cdot K^{T}}{\sqrt{d_{k}}}\right) \end{array} \end{array}$$

Finally, A was assigned to each V to calculate the output (O). Then the output of CLS in O was used as the input to the fully connected network, followed by the softmax function to obtain the probabilities of cell types, i.e.

(11) $$\begin{array}{c} \begin{array}{c} O=A\cdot V\\ p=\text{softmax}\left(W^{p}\cdot \mathrm{CLS}\right) \end{array} \end{array}$$

### 2.7 Clustering and identifying signature attentions of subclusters

For clustering, following the experimental setup of Chen *et al.* (2023), we first normalized and logarithmized all cell representations, and then used them as inputs to principal component analysis (PCA). Next, we constructed a nearest-neighbor graph using the PCA matrix. Finally, subclusters were identified using the Louvain algorithm (Blondel *et al.* 2008) with the hyperparameter “resolution” being set to 0.3.

For the signature attentions of subclusters, we normalized the attention scores to 1e4 and logarithmized it, and then identified the signature attentions by *sc.tl.rank_genes_groups(method=“wilcoxon”)*.

### 2.8 Performance evaluation

For cell type annotation, in order to assess the performance of different annotation methods more comprehensively, we used metrics that average the results overall categories (i.e. Precision_macro, Recall_macro, and *F*1_macro) to assess models’ ability to recognize different cell types. However, since different cell types in the single-cell datasets are not balanced, the above metrics are likely to be ineffective in evaluating the algorithm’s predictions for the data as a whole. Therefore, we used the *F*1_micro score as a complementary evaluation metric.

For cell clustering, we used representative metrics to assess the validity of our sICTA, namely the normalized mutual information (NMI) (Pedregosa *et al.* 2011) and the adjusted rand index (ARI) (Hubert and Arabie 1985). The ARI and NMI metrics were calculated by the Python functions *adjusted_rand_score*() and *normalized_mutual_info_score*() from the scikit-learn library (Pedregosa *et al.* 2011), respectively.

## 3 Results

### 3.1 The structure of sICTA

In this study, we introduce sICTA, a novel computational pipeline for marker-based cell annotation. As shown in Fig. 1, sICTA consists of two key modules. The first module (Fig. 1a) is a pseudo-label generator. It combines the cell type specificity of marker genes and cell expression data to generate a probability distribution over cell types for each cell in the dataset, which is used to construct pseudo-labels later. The second module (Fig. 1b) is a downstream classifier pretrained on cell expression data and pseudo-labels. Then, the classifier is iteratively optimized through a self-training process to continuously improve the performance. A key insight of our approach is that existing marker-based cell annotation methods do not adequately utilize marker genes to guide cell assignment. Furthermore, prior methods based on heuristic linear prediction do not capture the relationship between cells and labels well. This intuition has inspired us to combine the self-training strategy and Transformer with powerful fitting capabilities, which work together to form a reinforcement loop to capture the complex dependencies between cell representations and labels, providing an effective framework for marker-based cell type annotation.

### 3.2 Comparing different relevance assessment strategies

**Figure 2.**
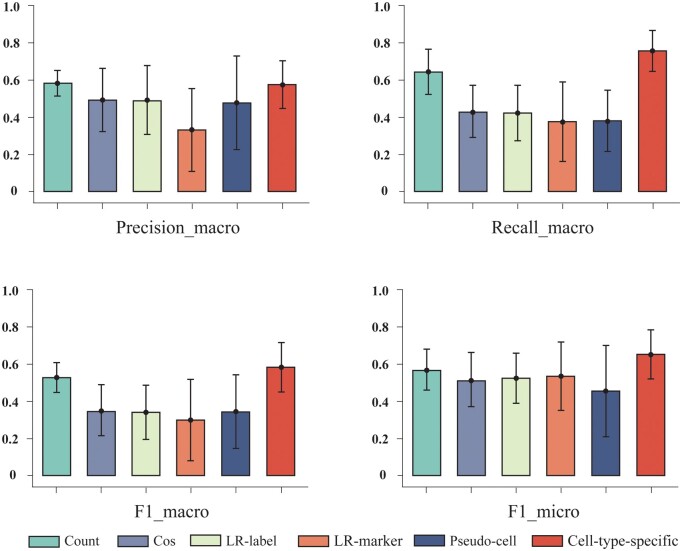
Average performance of different association assessment strategies on all datasets.

To generate pseudo-labels of higher quality, we quantify the performance of all association assessment strategies on 11 single-cell datasets. We used Precision_macro, Recall_macro, *F*1_macro, and *F*1_macro scores to comprehensively assess the performance of the models for cell annotation. The results are shown in Fig. 2 and Supplementary Table S2.

We discover that *Cell-type-specific* achieves the overall optimal performance. This may be due to its effective use of cell expression values and marker gene characterization, which can more accurately capture the relationship between cells and types. We find that the simple *Count* method achieves second best performance, which further reveals the importance of marker genes. In addition, the *pseudo-cell* strategy obtains overall lower and unstable performance, which may be due to the fact that on some datasets its generated cells differ significantly from the real data distribution. Interestingly, the strategies that incorporate external gene embeddings (i.e. *LR-label* and *LR-marker*) perform poorly, possibly due to the fact that the gene embeddings pretrained by previous work do not accurately map genes into the low-dimensional space. Therefore, in this work, we use the *Cell-type-specific* strategy to generate pseudo labels.

### 3.3 Providing high-quality cell type annotations

We benchmark sICTA on 11 scRNA-seq datasets compared with five SOTA marker-based single-cell annotation methods. Algorithmic and implementation details for sICTA and benchmarks are described in Materials and methods. We quantify the performance of all models by four evaluation metrics, and the results (Table 1) show that sICTA outperforms the baselines in the vast majority of the cases, with Precision_macro 9.1% higher, Recall_macro 12.8% higher, *F*1_macro 14.7% higher, and *F*1_micro 10.7% higher than the second best method. Compared to scType, sICTA shows an average improvement of 28.1% on all four metrics. A possible reason is that scType uses only linear heuristics for prediction, which are not fully accurate. Conversely, our sICTA leverages Transformer’s powerful nonlinear fitting capabilities, and therefore better captures potential dependencies between cells and types. We find that scSorter’s performance varies greatly across datasets, which may be due to the fact that its model tends to predict cells as classes with a high number of samples when faced with an unbalanced dataset. In contrast, *Std* shows the robustness of sICTA’s performance on different datasets. Furthermore, Supplementary Table S3 shows the standard deviation of the results from all methods affected by random seeds after five runs, and the standard deviation of sICTA is 0.006, which further demonstrates the stability of our methods.

**Table 1.** Performance of different methods on all datasets, where *Avg* represents the average of the performance overall single-cell datasets, **Bold** denotes the optimal result, and underline denotes the second best result. *Std* denotes the standard deviation of all single-cell datasets, and its smaller the better.

Model	**Avg** ↑	**Std** ↓	Muraro	Baron	Stoeckius	Zheng	Madissoon	Reyfman	Tirosh	Puram	Zeisel	Dominguez Conde	Yoshida
**Precision_macro**													
**scSorter**	0.580	0.254	0.927	0.942	0.562	0.610	0.269	0.448	0.678	0.065	0.826	0.519	0.536
**Garnett**	0.516	0.190	0.554	0.641	0.648	0.064	0.416	0.486	0.744	0.540	0.769	0.341	0.470
**SCINA**	0.627	0.164	0.859	0.796	0.742	0.645	0.535	0.516	0.830	0.342	0.702	0.481	0.452
**scType**	0.574	0.128	0.863	0.730	0.617	0.452	0.480	0.500	0.615	0.521	0.647	0.455	0.442
**MarkerCount**	0.736	**0.107**	0.830	0.750	0.794	0.794	0.680	0.709	0.826	0.782	0.843	0.472	**0.618**
**sICTA**	**0.807**	0.133	**0.942**	**0.961**	**0.949**	**0.828**	**0.860**	**0.738**	**0.831**	**0.798**	**0.852**	**0.572**	0.547
**Recall_macro**													
**scSorter**	0.564	0.249	0.949	0.947	0.449	0.562	0.300	0.395	0.416	0.167	**0.870**	0.572	0.582
**Garnett**	0.398	0.195	0.471	0.538	0.421	0.092	0.249	0.191	0.349	0.294	0.824	0.356	0.591
**SCINA**	0.654	0.170	0.859	0.790	0.871	0.689	0.594	0.538	0.891	0.387	0.626	0.496	0.453
**scType**	0.747	0.110	0.915	0.839	0.884	0.656	0.713	0.708	0.904	0.667	0.698	0.629	0.607
**MarkerCount**	0.729	0.128	0.861	0.585	0.859	**0.756**	0.680	0.704	0.925	0.800	0.775	0.530	0.541
**sICTA**	**0.822**	**0.106**	**0.968**	**0.963**	**0.907**	0.748	**0.808**	**0.749**	**0.928**	**0.802**	0.848	**0.694**	**0.632**
***F*1_macro**													
**scSorter**	0.483	0.287	0.936	0.942	0.419	0.204	0.175	0.302	0.449	0.092	**0.839**	0.469	0.481
**Garnett**	0.376	0.181	0.471	0.538	0.421	0.092	0.249	0.191	0.349	0.294	0.786	0.279	0.470
**SCINA**	0.568	0.165	0.842	0.701	0.754	0.479	0.479	0.441	0.774	0.340	0.604	0.448	0.391
**scType**	0.584	0.132	0.884	0.745	0.639	0.479	0.474	0.536	0.625	0.545	0.624	0.433	0.440
**MarkerCount**	0.680	0.138	0.806	0.480	0.821	**0.763**	0.631	0.676	0.808	0.788	0.760	0.440	0.502
**sICTA**	**0.785**	**0.133**	**0.954**	**0.960**	**0.926**	0.753	**0.786**	**0.705**	**0.810**	**0.792**	0.837	**0.575**	**0.537**
***F*1_micro**													
**scSorter**	0.644	0.234	0.949	0.952	0.720	0.722	0.526	0.177	**0.809**	0.384	0.874	0.551	0.505
**Garnett**	0.539	0.171	0.631	0.532	0.709	0.383	0.365	0.260	0.569	0.761	0.821	0.403	0.493
**SCINA**	0.620	0.189	0.900	0.793	0.895	0.798	0.531	0.338	0.623	0.421	0.564	0.531	0.426
**scType**	0.657	0.133	0.921	0.803	0.790	0.543	0.568	0.651	0.610	0.712	0.649	0.517	0.459
**MarkerCount**	0.754	0.148	0.795	0.476	0.942	**0.893**	0.690	0.780	0.754	0.937	0.843	0.525	**0.664**
**sICTA**	**0.835**	**0.122**	**0.967**	**0.968**	**0.947**	0.871	**0.758**	**0.812**	0.698	**0.967**	**0.886**	**0.712**	0.600

Next, we further refine the assessment of method performance. We show the prediction accuracies of sICTA and the top-performing benchmark model (i.e. MarkerCount) for each cell type on the different datasets (Supplementary Tables S4–14). The results show that sICTA achieves more accurate predictions on a wider range of cell types. In addition, we find that the number of different cell types in the dataset is unbalanced, i.e. the number of natural killer (NK) cells is about 107 times higher than that of Basal cells in the *Madissoon* dataset. Therefore, we further evaluate the performance of sICTA and MarkerCount on the rarest type in each dataset. The results (Supplementary Table S15) indicate that sICTA significantly outperforms MarkerCount in predicting rare types, which further demonstrates the robustness of our method. Moreover, we provide cluster-level evaluation for the clustering-based (i.e. scSorter, scType, and MarkerCount) methods, details of which can be found in Supplementary Note S2. Overall, we find that the cluster-level assessment achieves similar results to the cell-level assessment (Supplementary Tables S16 and S17). The MarkerCount method performs better than the other baselines in terms of both accuracy and coverage of clusters. Finally, we compared the running time of all methods on the *Muraro* dataset. The results (Supplementary Table S18) show that sICTA has similar run costs to scSorter, which alternately optimizes cluster assignment and cluster centroids, but is slower than models based on correlation assessment methods (i.e. SCINA and scType). We suggest that despite the speed disadvantage of sICTA, it offers a more promising route for cell type identification by combining neural network fitting capabilities with a self-training process.

### 3.4 Revealing effectiveness of different modules

**Figure 3.**
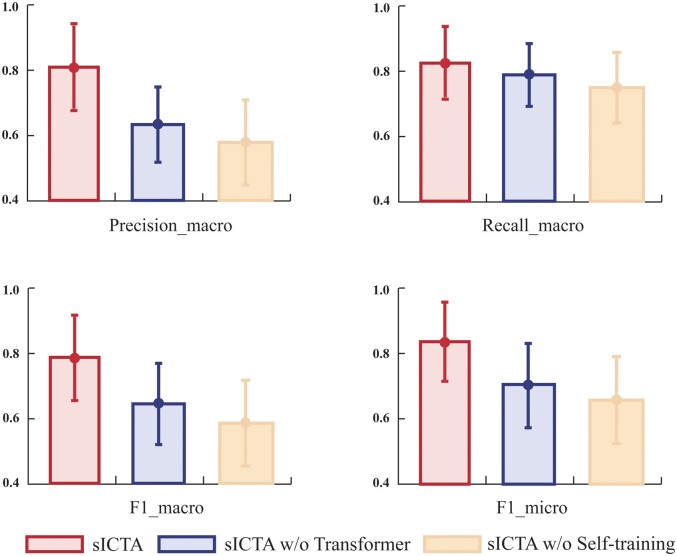
Ablation experiments of sICTA.

To further explore the performance of sICTA, we ablate the framework of sICTA and evaluate the ablated model on all datasets. Specifically, we evaluate the following cases: (i) Replacing Transformer with LR model (sICTA w/o Transformer) and (ii) Excluding the self-training strategy (sICTA w/o self-training). Figure 3 displays the average values of metrics across all datasets for each ablation case of sICTA.

**Figure 4.**
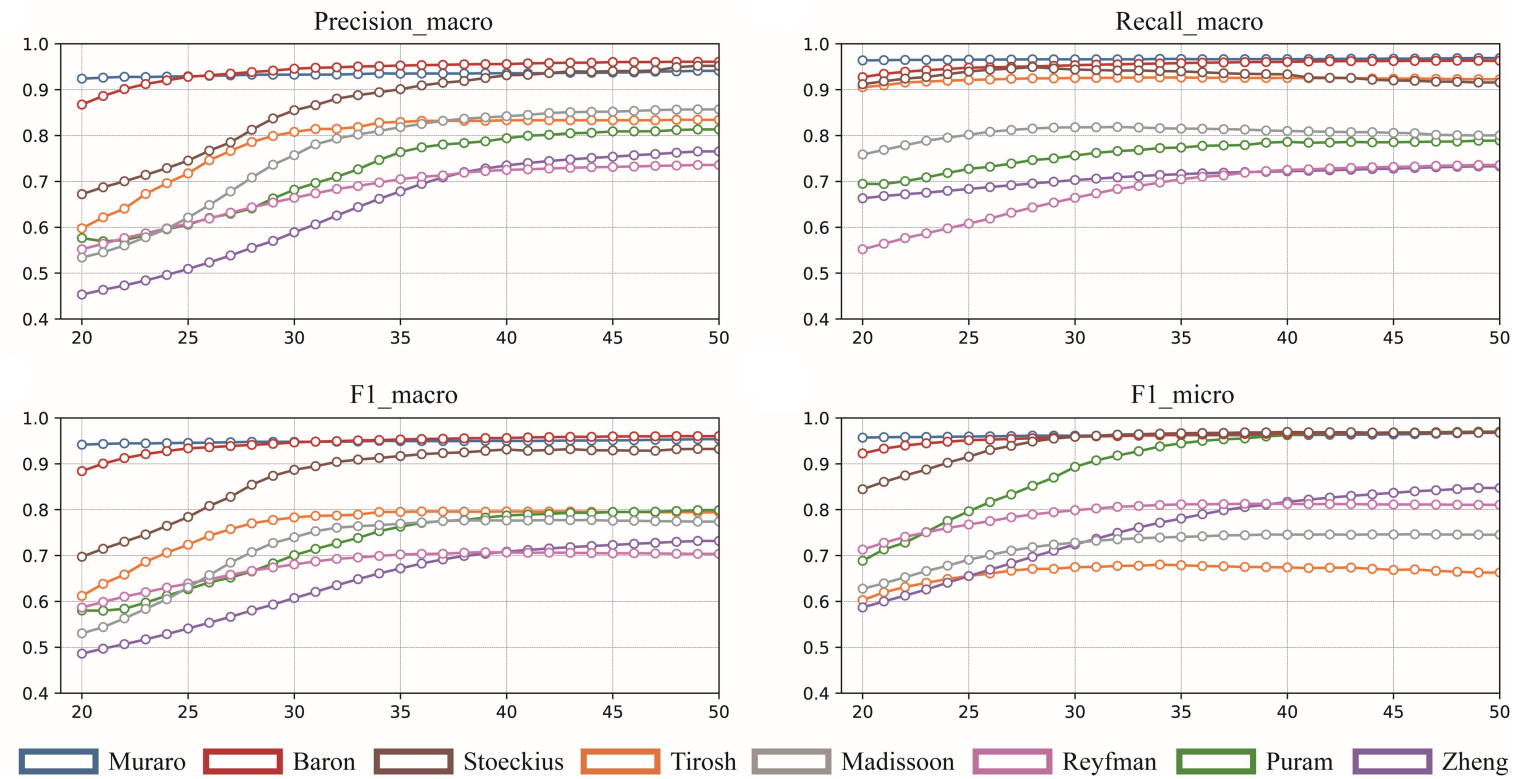
sICTA performance during self-training procedure. The horizontal coordinate is the epoch number.

The results show that the introduction of the self-training process significantly enhances the model’s ability in modeling single-cell data. The effectiveness of the self-training process is further demonstrated by the fact that it helped to improve the accuracy of the model even when the LR model was used as the downstream classifier. In addition, the performance of sICTA is improved by an average of 21.6% compared to sICTA w/o Transformer, which reveals the importance of nonlinear fitting ability. Next, we examine the changes in different metrics of sICTA during the self-training process, and the results on the 11 single-cell data consistently show that the performance of sICTA gradually improves until convergence during the self-training process, which proves the robustness of the model (Fig. 4 and Supplementary Fig. S1). More importantly, these findings also demonstrate that strong nonlinear fitting and the self-training process form a reinforcing loop that effectively optimizes the model and leads to better predictions. Interestingly, we also discover that the self-training process has relatively large differences in its impact on different datasets, such as *Tirosh* and *Muraro*. By Whitman *U*-test, we evaluate the difference in the performance of sICTA before and after self-training on all datasets by two metrics (*F*1-micro and *F*1-macro) that assess the overall performance. The results show that the performance after self-training exhibits a significant correlation with the accuracy of its initial pseudo-labels (*F*1-macro: *P*-value = .018 and *F*1-micro: *P*-value = .028), which demonstrates that the differences in self-training performance on different datasets are mainly caused by the noise level of the initial pseudo-labels.

### 3.5 Reflecting biological variability among different cell types

**Figure 5.**
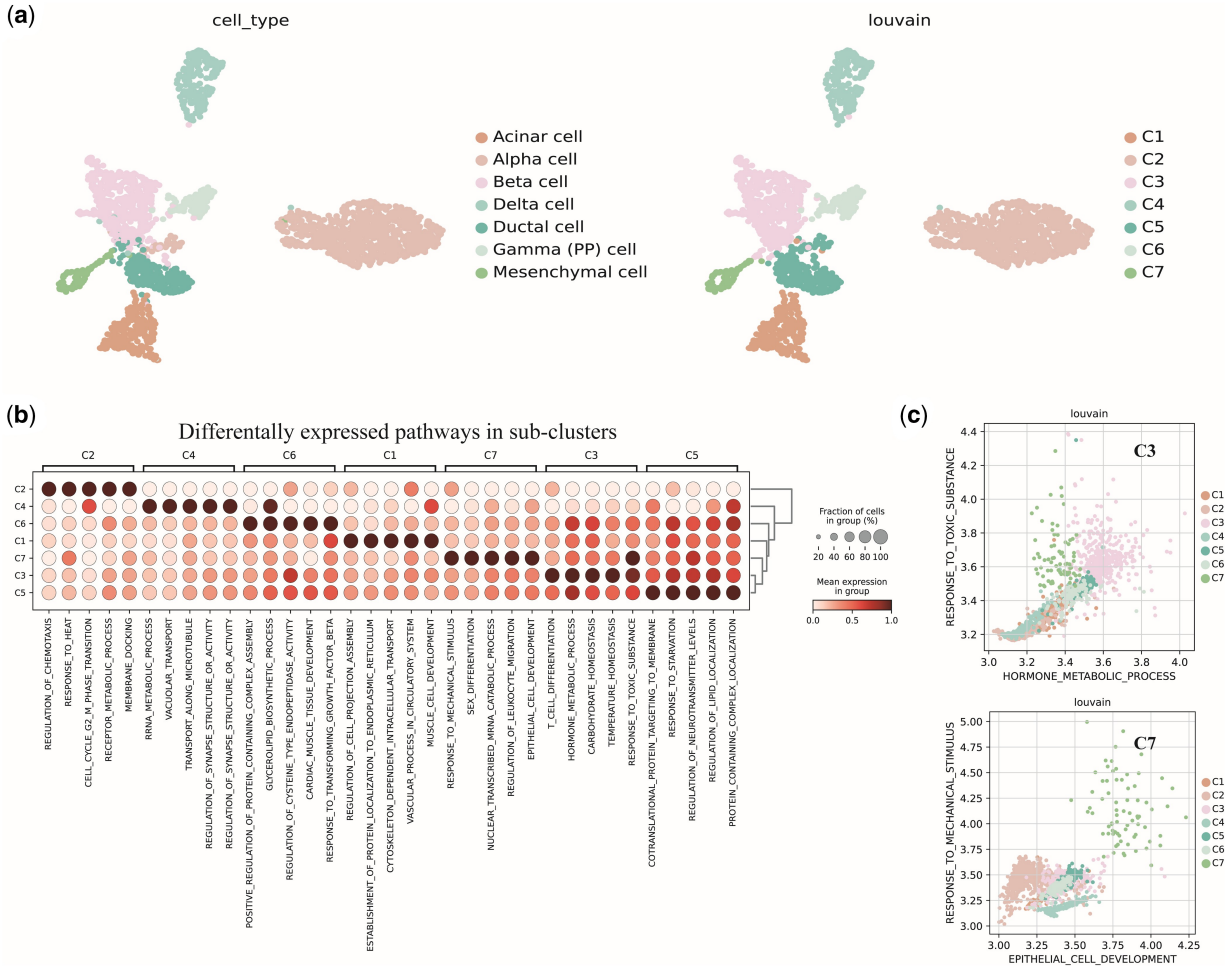
Attentional mechanisms provide interpretable annotations for biological insights. (a) UMAP visualization of sICTA embedding on pancreas data. (b) Differential expression analysis of pathway levels. (c) Different cell types are distinguished by the attention scores of the pathways. Each dot represents a cell and is colored by cell type.

Next, to verify the interpretability of the proposed sICTA, we apply it to the pancreas dataset (*Muraro*). We extract attention weights of class token (*CLS)* on pathways as low-dimensional features of cells and visualized them via uniform manifold approximation and projection (UMAP) algorithm (Fig. 5a). Notably, there is a clear separation among different cell types based on the information captured by the attention mechanism in sICTA. In addition, we quantify the clustering performance of the attention weights with the widely used metrics NMI and ARI, and the results (ARI = 0.902, NMI = 0.875) further demonstrate that the weights between cells and pathways captured by sICTA can reflect the biological variability among different cells.

Furthermore, we extract the differentially expressed pathways of each subpopulation. The results show that all isoforms have their own biological characteristics and potentially different functions and are closely related to the corresponding real cell types (Fig. 5b). For instance, sICTA detects cluster-specific pathways that separate Beta and Mesenchymal cells from other cells (Fig. 5c), respectively, which is consistent with previous observations (Paul Robertson and Harmon 2006, Landsman *et al.* 2011, Huang and Chang 2014, Carnevale *et al.* 2019). Moreover, we show the differential expression pathways captured by sICTA for each known cell type in the pancreas dataset. The Supplementary Table S19 lists the top three pathways captured for each cell type. Through an extensive literature search, we observe that 62% of the associations of pathways and cell types are consistent with previous experimental findings.

Overall, by analyzing the weights of attention, we find that sICTA is able to annotate key contributors to cell type assignment despite unconventional supervisory tasks, suggesting that it has the potential to reveal many new functional states of single cells and guide further experimental exploration.

## 4 Discussion

In this work, we have presented a marker-based cell type annotation model sICTA. We first evaluated six commonly used association assessment strategies to find high-quality pseudo-label generation strategies. The proposed sICTA achieved better *F*1 scores than the baseline methods in the benchmark analysis. Furthermore, comparison with the benchmark models and ablation analysis show that the self-training process and the model’s ability to capture nonlinear dependencies play important roles in cell type annotation. Moreover, by analyzing the attention matrix, we found that sICTA can effectively capture the biological signals behind the single-cell data, which provides a promising way to elucidate the underlying biological mechanisms.

In the future, we will explore in several directions. Firstly, although sICTA has achieved excellent performance, an ideal annotator should also be able to successfully identify unknown cell clusters, thus helping to provide further biological insights (Kim *et al.* 2022). Therefore, how to make unknown type predictions better benefit from high-quality annotation of known cell types is a valuable and challenging problem. Besides, there has been a recent influx of large models in the single-cell field (Cui *et al.* 2024, Theodoris *et al.* 2023), and it is also interesting to see how high-quality external knowledge from large models can be utilized. Moreover, although *Cell-type-specific* achieves the optimal overall performance on pseudo-label generation, we find that on some datasets (Supplementary Table S1), its performance is rather lower than that of the *Count* method. This may be due to the incomplete maker information of the dataset and the uneven distribution of data across different types, and how to generate more contextualized and high-quality pseudo-labels by better combining the characteristics of the dataset is less researched at present. Besides, we can find that MarkerCount and scSorter achieve superior performance in some cases. Compared to other methods, they explicitly integrate cell-cell association information with clustering, which has been shown to be effective in guiding cell clustering (Mrabah *et al.* 2023). Finally, since cell types can have subtypes, the labeling of cells and their corresponding marker information can be expanded into hierarchies (Nguyen and Griss 2022, Lee *et al.* 2023). We can replace the linear fully connected prediction layer in our sICTA with Transformer’s decoder, which allows us to predict paths in hierarchical labeling trees. In this way, the model can utilize the structural information of the labels and benefit from the outstanding performance of the Transformer on the Seq2Seq task.

In conclusion, the proposed sICTA is a novel framework that combines self-training and Transformer and performs well in single-cell annotation tasks. Moreover, the interpretability of sICTA enhances its ability to comprehensively analyze single-cell data. We envision that sICTA will bring new mechanistic understanding to the analysis of scRNA-seq data.

## Author contributions

Conceptualization, Methodology, Formal analysis, Writing – original draft: Hegang Chen; Validation, Writing – original draft, review & editing: Yuyin Lu; Funding acquisition, Supervision, Writing – review & editing: Yanghui Rao.

## Supplementary Material

btae569_Supplementary_Data

## References

[btae569-B1] Alquicira-Hernandez J , Sathe A, Ji HP et al scPred: accurate supervised method for cell-type classification from single-cell RNA-seq data. Genome Biol 2019;20:264. 31829268 10.1186/s13059-019-1862-5 PMC6907144

[btae569-B2] Aran D , Looney AP, Liu L et al Reference-based analysis of lung single-cell sequencing reveals a transitional profibrotic macrophage. Nat Immunol 2019;20:163–72. 30643263 10.1038/s41590-018-0276-y PMC6340744

[btae569-B3] Blondel VD , Guillaume J-L, Lambiotte R et al Fast unfolding of communities in large networks. J Stat Mech 2008;2008:P10008.

[btae569-B4] Carnevale I , Capula M, Giovannetti E et al A mechanical memory of pancreatic cancer cells. bioRxiv, 2019, preprint: not peer reviewed. 10.1101/730960

[btae569-B5] Chen J , Xu H, Tao W et al Transformer for one stop interpretable cell type annotation. Nat Commun 2023;14:223. 36641532 10.1038/s41467-023-35923-4 PMC9840170

[btae569-B6] Cui H , Wang C, Maan H et al scGPT: Toward building a foundation model for single-cell multi-omics using generative AI. Nat Methods 2024;21:1470–80. 38409223 10.1038/s41592-024-02201-0

[btae569-B7] Domínguez Conde C , Xu C, Jarvis LB et al Cross-tissue immune cell analysis reveals tissue-specific features in humans. Science 2022;376:eabl5197. 35549406 10.1126/science.abl5197 PMC7612735

[btae569-B8] Du J , Jia P, Dai Y et al Gene2vec: distributed representation of genes based on co-expression. BMC Genomics 2019;20:82. 30712510 10.1186/s12864-018-5370-x PMC6360648

[btae569-B9] Fawkner-Corbett D , Antanaviciute A, Parikh K et al Spatiotemporal analysis of human intestinal development at single-cell resolution. Cell 2021;184:810–26.e23. 33406409 10.1016/j.cell.2020.12.016 PMC7864098

[btae569-B10] Franzén O , Gan L-M, Björkegren JLM. PanglaoDB: a web server for exploration of mouse and human single-cell RNA sequencing data. Database 2019;2019:baz046. 30951143 10.1093/database/baz046 PMC6450036

[btae569-B11] Guo H , Li J. scSorter: assigning cells to known cell types according to marker genes. Genome Biol 2021;22:69. 33618746 10.1186/s13059-021-02281-7 PMC7898451

[btae569-B12] Huang Y , Chang Y. Regulation of pancreatic islet beta-cell mass by growth factor and hormone signaling. Prog Mol Biol Transl Sci 2014;121:321–49. 24373242 10.1016/B978-0-12-800101-1.00010-7

[btae569-B13] Hubert L , Arabie P. Comparing partitions. J Classif 1985;2:193–218.

[btae569-B14] Ianevski A , Giri AK, Aittokallio T. Fully-automated and ultra-fast cell-type identification using specific marker combinations from single-cell transcriptomic data. Nat Commun 2022;13:1246. 35273156 10.1038/s41467-022-28803-w PMC8913782

[btae569-B15] Jia S , Lysenko A, Boroevich KA et al scDeepInsight: a supervised cell-type identification method for scRNA-seq data with deep learning. Brief Bioinform 2023;24:bbad266. 37523217 10.1093/bib/bbad266 PMC10516353

[btae569-B16] Kim H , Lee J, Kang K et al MarkerCount: a stable, count-based cell type identifier for single-cell RNA-seq experiments. Comput Struct Biotechnol J 2022;20:3120–32. 35782735 10.1016/j.csbj.2022.06.010 PMC9233224

[btae569-B17] Kiselev VY , Yiu A, Hemberg M. scmap: projection of single-cell RNA-seq data across data sets. Nat Methods 2018;15:359–62. 29608555 10.1038/nmeth.4644

[btae569-B18] Landsman L , Nijagal A, Whitchurch TJ et al Pancreatic mesenchyme regulates epithelial organogenesis throughout development. PLoS Biol 2011;9:e1001143. 21909240 10.1371/journal.pbio.1001143 PMC3167782

[btae569-B19] Lee D-H. Pseudo-label: the simple and efficient semi-supervised learning method for deep neural networks. In: *ICML*, Atlanta, GA. 2013, 896.

[btae569-B20] Lee J , Kim M, Kang K et al Hierarchical cell-type identifier accurately distinguishes immune-cell subtypes enabling precise profiling of tissue microenvironment with single-cell RNA-sequencing. Brief Bioinform 2023;24:bbad006. 36681937 10.1093/bib/bbad006 PMC10025442

[btae569-B21] Lewinsohn DP , Vigh-Conrad KA, Conrad DF et al Consensus label propagation with graph convolutional networks for single-cell RNA sequencing cell type annotation. Bioinformatics 2023;39:btad360. 37267208 10.1093/bioinformatics/btad360 PMC10272704

[btae569-B22] Liu X , Tan JP, Schröder J et al Modelling human blastocysts by reprogramming fibroblasts into iBlastoids. Nature 2021;591:627–32. 33731926 10.1038/s41586-021-03372-y

[btae569-B23] Lubeck E , Cai L. Single-cell systems biology by super-resolution imaging and combinatorial labeling. Nat Methods 2012;9:743–8. 22660740 10.1038/nmeth.2069 PMC3418883

[btae569-B24] Maestre-Batlle D , Pena OM, Hirota JA et al Novel flow cytometry approach to identify bronchial epithelial cells from healthy human airways. Sci Rep 2017;7:42214. 28165060 10.1038/srep42214 PMC5292697

[btae569-B25] Meng Y , Shen J, Zhang C et al Weakly-supervised neural text classification. In: *CIKM*, Torino, Italy. ACM, 2018, 983–92.

[btae569-B26] Mikolajewicz N , Gacesa R, Aguilera-Uribe M et al Multi-level cellular and functional annotation of single-cell transcriptomes using scPipeline. Commun Biol 2022;5:1142. 36307536 10.1038/s42003-022-04093-2 PMC9616830

[btae569-B27] Mrabah N , Amar MM, Bouguessa M et al Toward convex manifolds: a geometric perspective for deep graph clustering of single-cell RNA-seq data. In: *IJCAI*, Macao, SAR, China. ijcai.org, 2023, 4855–63.

[btae569-B28] Nguyen V , Griss J. scAnnotatR: framework to accurately classify cell types in single-cell RNA-sequencing data. BMC Bioinformatics 2022;23:44. 35038984 10.1186/s12859-022-04574-5 PMC8762856

[btae569-B29] Paul Robertson R , Harmon JS. Diabetes, glucose toxicity, and oxidative stress: a case of double jeopardy for the pancreatic islet β cell. Free Radic Biol Med 2006;41:177–84. 16814095 10.1016/j.freeradbiomed.2005.04.030

[btae569-B30] Pedregosa F , Varoquaux G, Gramfort A et al scikit-learn: machine learning in Python. J Mach Learn Res 2011;12:2825–30.

[btae569-B31] Pliner HA , Shendure J, Trapnell C. Supervised classification enables rapid annotation of cell atlases. Nat Methods 2019;16:983–6. 31501545 10.1038/s41592-019-0535-3 PMC6791524

[btae569-B32] Rosenberg C , Hebert M, Schneiderman H. Semi-supervised self-training of object detection models. In: *WACV*, Colorado. IEEE Computer Society, 2005.

[btae569-B33] Shao X , Liao J, Lu X et al scCATCH: automatic annotation on cell types of clusters from single-cell RNA sequencing data. Iscience 2020;23:100882. 32062421 10.1016/j.isci.2020.100882 PMC7031312

[btae569-B34] Subramanian A , Tamayo P, Mootha VK et al Gene set enrichment analysis: a knowledge-based approach for interpreting genome-wide expression profiles. Proc Natl Acad Sci U S A 2005;102:15545–50. 16199517 10.1073/pnas.0506580102 PMC1239896

[btae569-B35] Theodoris CV , Xiao L, Chopra A et al Transfer learning enables predictions in network biology. Nature 2023;618:616–24. 37258680 10.1038/s41586-023-06139-9 PMC10949956

[btae569-B36] Xu J , Zhang A, Liu F et al CiForm as a transformer-based model for cell-type annotation of large-scale single-cell RNA-seq data. Brief Bioinform 2023;24:bbad195. 37200157 10.1093/bib/bbad195

[btae569-B37] Zeisel A , Muñoz-Manchado AB, Codeluppi S et al Cell types in the mouse cortex and hippocampus revealed by single-cell RNA-seq. Science 2015;347:1138–42. 25700174 10.1126/science.aaa1934

[btae569-B38] Zhang X , Lan Y, Xu J et al CellMarker: a manually curated resource of cell markers in human and mouse. Nucleic Acids Res2019a;47:D721–8. 30289549 10.1093/nar/gky900 PMC6323899

[btae569-B39] Zhang Z , Luo D, Zhong X et al SCINA: a semi-supervised subtyping algorithm of single cells and bulk samples. Genes (Basel) 2019b;10:531. 31336988 10.3390/genes10070531 PMC6678337

