# Supplementary material for: A self-training interpretable cell type annotation framework using specific marker gene
Source: Bioinformatics. 2024 Sep 23;40(10):btae569. doi: 10.1093/bioinformatics/btae569 (PMC11488977; doi:10.1093/bioinformatics/btae569)
Supplement: btae569_Supplementary_Data [file btae569_supplementary_data.zip › sICTA_Supplementary_0919.pdf]

SUPPLEMENTARY INFORMATION FOR

A self-training interpretable cell type annotation framework using specific marker gene

Hegang Chen[1], Yuyin Lu[1] and Yanghui Rao[1*]

[1]School of Computer Science and Engineering, Sun Yat-sen University, Guangzhou, China

*Correspondence author: raoyangh@mail.sysu.edu.cn

Supplementary Figures

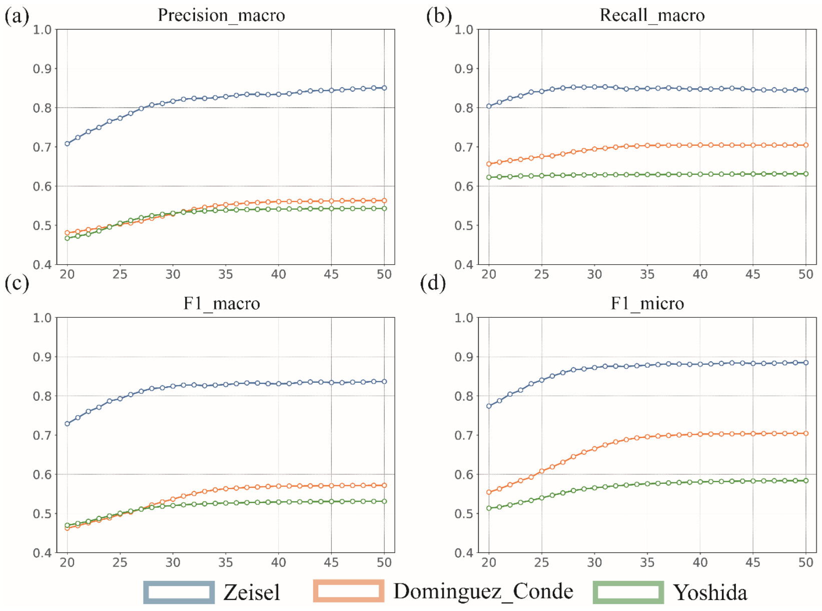

Supplementary Fig. 1. sICTA performance during self-training procedure. The horizontal coordinate is the epoch number.

Supplementary Notes

Supplementary Note 1: Hyparameter settings

Our sICTA is implemented in Python, and the core model is built on the Pytorch (v.1.12.1) framework. sICTA first generates pseudo-labels using the **Cell-type-specific** strategy and pre-trains downstream classifiers based on this. Next, we iteratively optimise the downstream classifiers with the self-training strategy until the model converges. The downstream classifier used in this work contains a Transformer layer and a fully connected layer. We set the depth of the Transformer architecture to 2 and the number of attention heads to 4. In addition, all embedding dimensions in sICTA are set to 100. Throughout the training process, the batch size is set to 128, the pre-training phase costs 20 epochs, the self-training phase costs 30 epochs for a total of 50 epochs, and the optimization of sICTA is done by Adam. All experiments are performed using publicly available modeling code and in a Python environment equipped with an Nvidia RTX 1080-Ti GPU and 128G RAM. Moreover, for the fairness of the comparison, the same marker genes are used for all methods. It is worth noting that for all models, they are categorised into methods affected by random seeds (sICTA and Garnett) and methods not affected by random seeds (scSorter, SCINA, MarkerCount and scType) by examining their papers and published codes. For all methods affected by random seeds, we report their average results over five runs.

Supplementary Note 2: Performance evaluation at the cluster level

The performance evaluation at the cluster level consists of the following 2 parts:

1) **Prediction accuracy of clusters:** According to the majority voting strategy, we set the true cell type of a cluster as the cell type that accounts for the most cells in that cluster, and calculate the prediction accuracy of clusters by comparing the predicted types of the clusters to their true types.
2) **Cluster coverage for real cell types:** We assess the percentage of cell types covered by clusters with accurate predictions.

It is worth noting that scSorter does not explicitly utilise a clustering algorithm; instead, it defines an objective function similar to the *K*-means clustering algorithm and generates the number of cluster centres based on known marker information. Therefore, we consider the predicted types as clusters in order to evaluate its performance at the cluster level.

Supplementary Tables

Supplementary Table 1. Description of all single-cell datasets.

Dataset	Tissue	#Cell	#Class	Protocol	Accession ID	Reference
**Muraro**	Human pancreas	2,098	7	CEL-Seq2	GSE84133	Muraro (Muraro *et al.*, 2016)
**Baron**	Human pancreas	7,742	6	CEL-Seq2	GSE85241	Baron (Baron *et al.*, 2016)
**Stoeckius**	Human blood	7,467	6	Drop-seq	GSE100866	Stoeckius (Stoeckius *et al.*, 2017)
**Zheng**	Mouse blood	68,302	6	10X genomics	GSE93421	Zheng (Zheng *et al.*, 2017)
**Madissoon**	Human lung	24,662	11	10X v2	ERP114453	Madissoon (Madissoon *et al.*, 2020)
**Reyfman**	Human lung	34,393	12	10X v2	SRP218543	Reyfman (Reyfman *et al.*, 2019)
**Tirosh**	Human tumor (Melanoma)	2,949	6	Smart-Seq2	GSE72056	Tirosh (Tirosh *et al.*, 2016)
**Puram**	Human tumor (HeadNeck)	3,224	6	Smart-Seq2	GSE103322	Puram (Puram *et al.*, 2017)
**Zeisel**	Mouse brain	3,005	7	STRT-Seq	GSE60361	Zeisel (Zeisel *et al.*, 2015)
**Dominguez-Conde**	Human blood	25,362	9	10X genomics	E-MTAB-11536	Dominguez-Conde (Domínguez Conde *et al.*, 2022)
**Yoshida**	Human blood	43,468	14	10X genomics	GSE168215	Yoshida(Yoshida *et al.*, 2022)

Supplementary Table 2. Performance of different association assessment strategies, where **Avg** represents the average of the performance over all single-cell datasets, **Bold** denotes the optimal result, and underline denotes the second best result. **Std** denotes the standard deviation of all single-cell datasets, and its smaller the better.

Model	Avg	Std	Muraro	Baron	Stoeckius	Zheng	Madissoon	Reyfman	Tirosh	Puram	Zeisel	Dominguez-Conde	Yoshida
**Precision_macro**													
**Count**	**0.582**	**0.069**	0.660	0.613	0.647	**0.610**	0.560	0.619	0.598	0.520	0.638	0.421	**0.513**
**Cos**	0.491	0.170	0.554	0.641	0.648	0.064	0.416	0.486	0.744	0.540	0.381	**0.476**	0.451
**LR-label**	0.487	0.185	0.572	0.644	0.641	0.064	0.229	0.473	0.477	0.725	0.621	0.463	0.449
**LR-marker**	0.329	0.223	0.573	0.701	0.122	0.064	0.135	0.123	0.241	0.143	0.621	0.462	0.438
**Pseudo-cell**	0.575	0.252	0.426	0.192	**0.721**	0.563	**0.576**	**0.638**	**0.824**	**0.780**	0.267	0.117	0.124
**Cell-type-specific**	0.575	0.128	**0.863**	**0.730**	0.615	0.521	0.480	0.500	0.617	0.452	**0.647**	0.455	0.442
**Recall_macro**													
**Count**	0.545	**0.071**	0.410	0.489	0.659	0.612	0.534	0.548	0.612	0.479	0.626	0.518	0.510
**Cos**	0.376	0.145	0.471	0.538	0.421	0.092	0.249	0.191	0.349	0.294	0.537	0.525	0.468
**LR-label**	0.422	0.149	0.519	0.659	0.392	0.167	0.332	0.306	0.363	0.251	0.619	0.538	0.498
**LR-marker**	0.376	0.214	0.478	0.813	0.167	0.167	0.306	0.185	0.169	0.200	0.619	0.539	0.488
**Pseudo-cell**	0.376	0.164	0.423	0.246	0.696	0.474	0.372	0.415	0.518	0.457	0.150	0.296	0.092
**Cell-type-specific**	**0.747**	0.110	**0.915**	**0.840**	**0.904**	**0.667**	**0.713**	**0.708**	**0.884**	**0.655**	**0.698**	**0.629**	**0.607**
**F1_macro**													
**Count**	0.526	**0.081**	0.410	0.489	**0.659**	**0.612**	**0.534**	**0.548**	0.612	0.479	0.584	0.408	**0.453**
**Cos**	0.344	0.123	0.471	0.538	0.421	0.092	0.249	0.191	0.349	0.294	0.402	0.409	0.368
**LR-label**	0.340	0.146	0.432	0.525	0.332	0.092	0.154	0.207	0.348	0.251	0.590	0.425	0.379
**LR-marker**	0.297	0.220	0.440	**0.745**	0.141	0.092	0.076	0.115	0.106	0.166	0.588	0.424	0.376
**Pseudo-cell**	0.337	0.190	0.362	0.214	0.606	0.490	0.298	0.390	0.566	**0.523**	0.117	0.107	0.031
**Cell-type-specific**	**0.582**	0.133	**0.884**	**0.745**	0.625	0.525	0.474	0.536	**0.639**	0.479	**0.624**	**0.433**	0.440
**F1_micro**													
**Count**	0.565	**0.110**	0.377	0.552	0.565	**0.727**	**0.581**	**0.667**	0.729	0.430	0.625	0.486	**0.483**
**Cos**	0.509	0.146	0.631	0.532	0.709	0.383	0.365	0.260	0.569	0.761	0.406	0.529	0.449
**LR-label**	0.523	0.135	0.590	0.530	0.668	0.383	0.383	0.255	0.564	0.742	0.624	**0.554**	0.462
**LR-marker**	0.534	0.183	0.616	**0.845**	0.730	0.383	0.285	0.234	0.443	0.712	0.623	0.551	0.453
**Pseudo-cell**	0.454	0.246	0.469	0.275	**0.828**	0.623	0.420	0.528	0.628	**0.802**	0.212	0.175	0.038
**Cell-type-specific**	**0.651**	0.131	**0.921**	0.803	0.610	0.653	0.568	0.652	**0.790**	0.543	**0.649**	0.517	0.459

Supplementary Table 3. Standard deviation of 5 replications of methods affected by random seeds, where avg represents the average of the standard deviation over all single-cell datasets.

Model	Avg	Muraro	Baron	Stoeckius	Zheng	Madissoon	Reyfman	Tirosh	Puram	Zeisel	Dominguez-Conde	Yoshida
**Precision_macro**												
**Garnett**	0.009	0.009	0.011	0.011	**0.013**	0.011	0.011	0.011	**0.001**	**0.014**	**0.001**	**0.002**
**sICTA**	**0.007**	**0.001**	**0.001**	**0.003**	0.020	**0.005**	**0.004**	**0.003**	0.014	0.015	0.005	0.004
**Recall_macro**												
**Garnett**	0.010	0.006	0.021	0.010	0.013	0.010	0.012	0.008	**0.011**	0.014	**0.001**	0.008
**sICTA**	**0.006**	**0.001**	**0.001**	**0.009**	**0.006**	**0.002**	**0.004**	**0.004**	0.019	**0.013**	0.006	**0.005**
**F1_macro**												
**Garnett**	0.011	0.008	0.030	0.007	0.020	0.007	0.015	0.009	**0.004**	0.016	**0.001**	0.008
**sICTA**	**0.005**	**0.001**	**0.001**	**0.006**	**0.005**	**0.006**	**0.003**	**0.004**	0.008	**0.014**	0.005	**0.007**
**F1_micro**												
**Garnett**	0.013	0.007	0.026	0.007	0.034	0.015	0.022	0.004	**0.004**	0.012	**0.001**	0.015
**sICTA**	**0.005**	**0.001**	**0.001**	**0.001**	**0.014**	**0.006**	**0.002**	**0.002**	**0.004**	**0.007**	0.005	**0.014**

Supplementary Table 4. Prediction accuracy of different cell types on the Muraro dataset.

Model	Beta cell (21.4%)	Acinar cell (10.4)	Delta cell (9.2%)	Ductal cell (11.7%)	Mesenchymal cell (3.8%)	Gamma (PP) cell (4.8%)	Alpha cell (38.7%)
**MarkerCount**	0.919	0.872	0.968	0.024	0	0	**0.979**
**sICTA**	**0.946**	**0.990**	**0.995**	**0.898**	**0.988**	**0.980**	0.970

Supplementary Table 5. Prediction accuracy of different cell types on the Baron dataset.

Model	Alpha cell (30.0%)	Delta cell (7.8%)	Beta cell (32.6%)	Ductal cell (13.9%)	Acinar cell (12.4%)	Gamma (PP) cell (3.3%)
**MarkerCount**	0.936	0.886	0.967	0.623	0.912	0.545
**sICTA**	**0.987**	**0.973**	**0.986**	**0.828**	**0.994**	**0.988**

Supplementary Table 6. Prediction accuracy of different cell types on the Tirosh dataset.

Model	T cell (73.0%)	B cell (17.4%)	Macrophage (4.0%)	Endothelial cell (2.1%)	NK cell (1.6%)	Fibroblast (1.9%)
**MarkerCount**	**0.837**	0.994	**1**	**1**	0.913	0.553
**sICTA**	0.577	**0.998**	**1**	**1**	**1**	**1**

Supplementary Table 7. Prediction accuracy of different cell types on the Puram dataset.

Model	Fibroblast (44.7%)	T cell (38.4%)	Dendritic cell (1.6%)	Macrophage (3.0%)	Endothelial cell (8.1%)	B cell (4.2%)
**MarkerCount**	0.918	**0.993**	0	0.755	**0.965**	0.927
**sICTA**	**0.992**	**0.993**	0	**0.785**	0.938	**0.978**

Supplementary Table 8. Prediction accuracy of different cell types on the Madissoon dataset.

Model	B cell (3.1%)	Ciliated cell (0.9%)	T cell (27.4%)	Macrophage (11.2%)	Alveolar Type2 (9.8%)	Monocyte (12.3%)	Secretory cell (0.4%)	Basal cell (0.2%)	NK cell (21.4%)	Dendritic cell (3.9%)	Fibroblast (9.4%)
**MarkerCount**	0.913	**0.991**	0.588	0.883	0.040	**0.472**	**0.933**	0.933	**0.880**	0.200	0.855
**sICTA**	**0.953**	0.961	**0.694**	**0.994**	**0.972**	0.119	0.900	**0.978**	0.823	**0.510**	**0.979**

Supplementary Table 9. Prediction accuracy of different cell types on the Reyfman dataset.

Model	T cell (7.1%)	Fibroblast (1.9%)	Ciliated cell (15.9%)	B cell (1.0%)	Epithelial cell (0.3%)	NK cell (2.3%)	Alveolar Type2 (10.4%)	Monocyte (6.7%)	Dendritic cell (2.1%)	Endothelial cell (10.3%)	Macrophage (40.0%)	Basal cell (2.0%)
**MarkerCount**	**0.899**	0.981	**0.997**	0.634	**0.603**	0.758	0	**0.448**	0.292	0.813	0.925	0.931
**sICTA**	0.794	**0.989**	**0.997**	**0.932**	0.526	**0.867**	0	0.240	**0.641**	**0.994**	**0.980**	**0.970**

Supplementary Table 10. Prediction accuracy of different cell types on the Stoeckius dataset.

Model	T cell (44.3%)	NK cell (14.5%)	Monocyte (33.8%)	Megakaryocyte (1.2%)	Dendritic cell (1.6%)	B cell (4.6%)
**MarkerCount**	0.994	**0.981**	0.919	0.295	0.857	0.953
**sICTA**	**0.999**	0.873	**0.976**	**0.841**	**0.958**	**0.976**

Supplementary Table 11. Prediction accuracy of different cell types on the Zheng dataset.

Model	T cell (71.2%)	B cell (8.6%)	NK cell (12.8%)	Monocyte (4.2%)	Dendritic cell (3.2%)
**MarkerCount**	**0.942**	**0.642**	**0.928**	**0.832**	0.418
**sICTA**	0.906	0.614	0.835	0.696	**0.486**

Supplementary Table 12. Prediction accuracy of different cell types on the Zeisel dataset.

Model	endothelial-mural (7.8%)	oligodendrocytes (27.3%)	pyramidal CA1 (31.2%)	pyramidal SS (13.3%)	interneurons (9.7%)	microglia (3.3%)
**MarkerCount**	0.178	0.830	**0.973**	**0.935**	0.931	0.806
**sICTA**	**0.349**	**0.960**	0.928	0.882	**0.941**	**0.939**

Supplementary Table 13. Prediction accuracy of different cell types on the Dominguez_Conde dataset.

Model	Non-classical monocytes (0.8%)	Tcm/Naive helper T cells (27.1%)	Regulatory T cells (2.8%)	Tem/Effector helper T cells (17.4%)	Tem/Trm cytotoxic T cells (2.4%)	Tem/Temra cytotoxic T cells (6.9%)	Tcm/Naive cytotoxic T cells (3.8%)	Classical monocytes (23.2%)	CD16+ NK cells (15.4%)
**MarkerCount**	0.099	0.064	0.494	**0.521**	0.814	0.094	**0.977**	**0.983**	0.719
**sICTA**	**0.756**	**0.697**	**0.781**	0.409	**0.972**	**0.121**	0.805	0.969	**0.833**

Supplementary Table 14. Prediction accuracy of different cell types on the Yoshida dataset.

Model	Memory B cells (0.5%)	Naive B cells (5.6%)	Non-classical monocytes (2.7%)	Tcm/Naive helper T cells (28.0%)	DC2 (0.8%)	Megakaryocytes/platelets (1.1%)	Regulatory T cells (0.9%)
**MarkerCount**	0.161	**0.866**	0.202	**0.726**	0.762	0.995	0
**sICTA**	**0.339**	0.825	**0.720**	0.511	**0.966**	**1**	**0.697**

Model	Tem/Effector helper T cells (6.8%)	Tem/Trm cytotoxic T cells (2.7%)	Tem/Temra cytotoxic T cells (11.0%)	Tcm/Naive cytotoxic T cells (4.7%)	Classical monocytes (15.9%)	CD16+ NK cells (16.8%)	MAIT cells (2.6%)
**MarkerCount**	0.338	**0.720**	**0.148**	**0.876**	0.896	**0.876**	0.006
**sICTA**	**0.460**	0.718	0.139	0.568	**0.910**	0.608	**0.386**

Supplementary Table 15. Prediction accuracy of sICTA and MarkerCount on the rarest cell type in each single cell data. Percentages indicate the proportion of the rarest cell type in each dataset.

Model	Avg (1.8%)	Muraro (3.8%)	Baron (3.3%)	Stoeckius (1.2%)	Zheng (3.2%)	Madissoon (0.2%)	Reyfman (0.3%)	Tirosh (1.6%)	Puram (1.6%)	Zeisel (3.3%)	Dominguez-Conde (0.8%)	Yoshida (0.5%)
**MarkerCount**	0.433	0	0.545	0.295	0.418	0.933	**0.603**	0.913	**0**	0.806	0.099	0.161
**sICTA**	**0.713**	**0.988**	**0.988**	**0.841**	**0.486**	**0.978**	0.526	**1**	0	**0.939**	**0.756**	**0.339**

Supplementary Table 16. Predictive accuracy of clusters.

Model	Avg	Muraro	Baron	Stoeckius	Zheng	Madissoon	Reyfman	Tirosh	Puram	Zeisel	Dominguez-Conde	Yoshida
scSorter	0.711	1	1	1	0.800	0.363	0.500	1	0	1	0.667	0.500
scType	0.129	0	0.125	0.125	0.222	0.048	0.136	0.231	0.143	0.211	0.182	0
MarkerCount	0.812	0.901	0.682	1	0.946	0.705	0.815	0.851	0.964	0.852	0.570	0.654

Supplementary Table 17. Cluster coverage for real cell types.

Model	Avg	Muraro	Baron	Stoeckius	Zheng	Madissoon	Reyfman	Tirosh	Puram	Zeisel	Dominguez-Conde	Yoshida
scSorter	0.682	1	1	0.667	0.800	0.363	0.500	1	0	1	0.667	0.500
scType	0.217	0	0.333	0.333	0.4	0.091	0.166	0.167	0.167	0.286	0.444	0
MarkerCount	0.829	1	0.571	1	1	0.727	0.833	0.666	0.833	1	0.778	0.714

Supplementary Table 18. Running time of all methods on **Muraro** dataset.

Metric	scSorter	Garnett	SCINA	scType	MarkerCount	sICTA
**Speed**	138.1s	368.8s	**10.6s**	26.8	26.3s	132.1s

Supplementary Table 19. A total of 13 predictions (62% success rate) have been validation in previously reported work. NA: There is non-available literature for the prediction.

Cell_type	Pathways	Reference
Acinar cell	GOBP_REGULATION_OF_CELL_PROJECTION_ASSEMBLY	NA
	GOBP_VASCULAR_PROCESS_IN_CIRCULATORY_SYSTEM	(Cleaver and Dor, 2012)
	GOBP_POSITIVE_REGULATION_OF_PROTEIN_CONTAINING_COMPLEX_ASSEMBLY	(Michelle M *et al.*, 2020)
Alpha cell	GOBP_HEPATICOBILIARY_SYSTEM_DEVELOPMENT	NA
	GOBP_REGULATION_OF_CHEMOTAXIS	NA
	GOBP_CELL_CYCLE_G2_M_PHASE_TRANSITION	(Lu *et al.*, 2019)
Beta cell	GOBP_T_CELL_DIFFERENTIATION	(Crotty, 2015)
	GOBP_HORMONE_METABOLIC_PROCESS	(Deeney *et al.*, 2000)
	GOBP_CARBOHYDRATE_HOMEOSTASIS	(Acosta-Montaño 2018)
Delta cell	GOBP_RRNA_METABOLIC_PROCESS	NA
	GOBP_TRANSPORT_ALONG_MICROTUBULE	NA
	GOBP_REGULATION_OF_SYNAPSE_STRUCTURE_OR_ACTIVITY	NA
Ductal cell	GOBP_COTRANSLATIONAL_PROTEIN_TARGETING_TO_MEMBRANE	(Clegg *et al.*, 1999)
	GOBP_REGULATION_OF_NEUROTRANSMITTER_LEVELS	(Yang *et al.*, 2021)
	GOBP_RESPONSE_TO_STARVATION	(Tavano *et al.*, 2015)
PP cell	GOBP_POSITIVE_REGULATION_OF_PROTEIN_CONTAINING_COMPLEX_ASSEMBLY	(Meda, 2013)
	GOBP_RESPONSE_TO_TRANSFORMING_GROWTH_FACTOR_BETA	(Gentry, 1988)
	GOBP_CARDIAC_MUSCLE_TISSUE_DEVELOPMENT	NA
Mesenchymal cell	GOBP_SEX_DIFFERENTIATION	(Hughes, 2001)
	GOBP_NUCLEAR_TRANSCRIBED_MRNA_CATABOLIC_PROCESS	NA
	GOBP_EPITHELIAL_CELL_DEVELOPMENT	(Thiery, 2003)

References

Acosta-Montaño,P. and García-González,V. (2018) Effects of Dietary Fatty Acids in Pancreatic Beta Cell Metabolism, Implications in Homeostasis. *Nutrients*, **10**, 393.

Baron,M. *et al.* (2016) A Single-Cell Transcriptomic Map of the Human and Mouse Pancreas Reveals Inter- and Intra-cell Population Structure. *Cell Systems*, **3**, 346-360.e4.

Cleaver,O. and Dor,Y. (2012) Vascular instruction of pancreas development. *Development*, **139**, 2833–2843.

Clegg,R.A. *et al.* (1999) Expression of enzymes of covalent protein modification during regulated and dysregulated proliferation of mammary epithelial cells: PKA, PKC and NMT. *Advances in Enzyme Regulation*, **39**, 175–203.

Crotty,S. (2015) A brief history of T cell help to B cells. *Nat Rev Immunol*, **15**, 185–189.

Deeney,J.T. *et al.* (2000) Metabolic control ofβ-cell function. *Seminars in Cell & Developmental Biology*, **11**, 267–275.

Domínguez Conde,C. *et al.* (2022) Cross-tissue immune cell analysis reveals tissue-specific features in humans. *Science*, **376**, eabl5197.

Gentry,L.E. (1988) Molecular Events in the Processing of Recombinant Type 1 Pre-Pro-Transforming Growth Factor Beta to the Mature Polypeptide. **8**.

Hughes,I.A. (2001) Minireview: Sex Differentiation. *Endocrinology*.

Lu,C.-J. *et al.* (2019) Single-cell analyses identify distinct and intermediate states of zebrafish pancreatic islet development. *Journal of Molecular Cell Biology*, **11**, 435–447.

Madissoon,E. *et al.* (2020) scRNA-seq assessment of the human lung, spleen, and esophagus tissue stability after cold preservation. *Genome Biol*, **21**, 1.

Meda,P. (2013) Protein-Mediated Interactions of Pancreatic Islet Cells. *Scientifica*, **2013**, 1–22.

Michelle M,C. *et al.* (2020) Pancreatic Acinar Cell Protein Synthesis, Intracellular Transport, and Export. *The Exocrine Pancreas Knowledge Base*.

Muraro,M.J. *et al.* (2016) A Single-Cell Transcriptome Atlas of the Human Pancreas. *Cell Systems*, **3**, 385–394.

Puram,S.V. *et al.* (2017) Single-Cell Transcriptomic Analysis of Primary and Metastatic Tumor Ecosystems in Head and Neck Cancer. *Cell*, **171**, 1611–1624.

Stoeckius,M. *et al.* (2017) Simultaneous epitope and transcriptome measurement in single cells. *Nat Methods*, **14**, 865–868.

Tavano,F. *et al.* (2015) SIRT1 and circadian gene expression in pancreatic ductal adenocarcinoma: Effect of starvation. *Chronobiology International*, **32**, 497–512.

Thiery,J.P. (2003) Epithelial–mesenchymal transitions in development and pathologies. *Current Opinion in Cell Biology*, **15**, 740–746.

Tirosh,I. *et al.* (2016) Dissecting the multicellular ecosystem of metastatic melanoma by single-cell RNA-seq. *Science*, **352**, 189–196.

Yang,X. *et al.* (2021) Pathophysiologic Role of Neurotransmitters in Digestive Diseases. *Front. Physiol.*, **12**, 567650.

Yoshida,M. *et al.* (2022) Local and systemic responses to SARS-CoV-2 infection in children and adults. *Nature*, **602**, 321–327.

Zeisel,A. *et al.* (2015) Cell types in the mouse cortex and hippocampus revealed by single-cell RNA-seq. *Science*, **347**, 1138–1142.

Zheng,G.X.Y. *et al.* (2017) Massively parallel digital transcriptional profiling of single cells. *Nat Commun*, **8**, 14049.
