# Supplementary figures and images for: A self-training interpretable cell type annotation framework using specific marker gene
Source: Bioinformatics. 2024 Sep 23;40(10):btae569. doi: 10.1093/bioinformatics/btae569 (PMC11488977; doi:10.1093/bioinformatics/btae569)

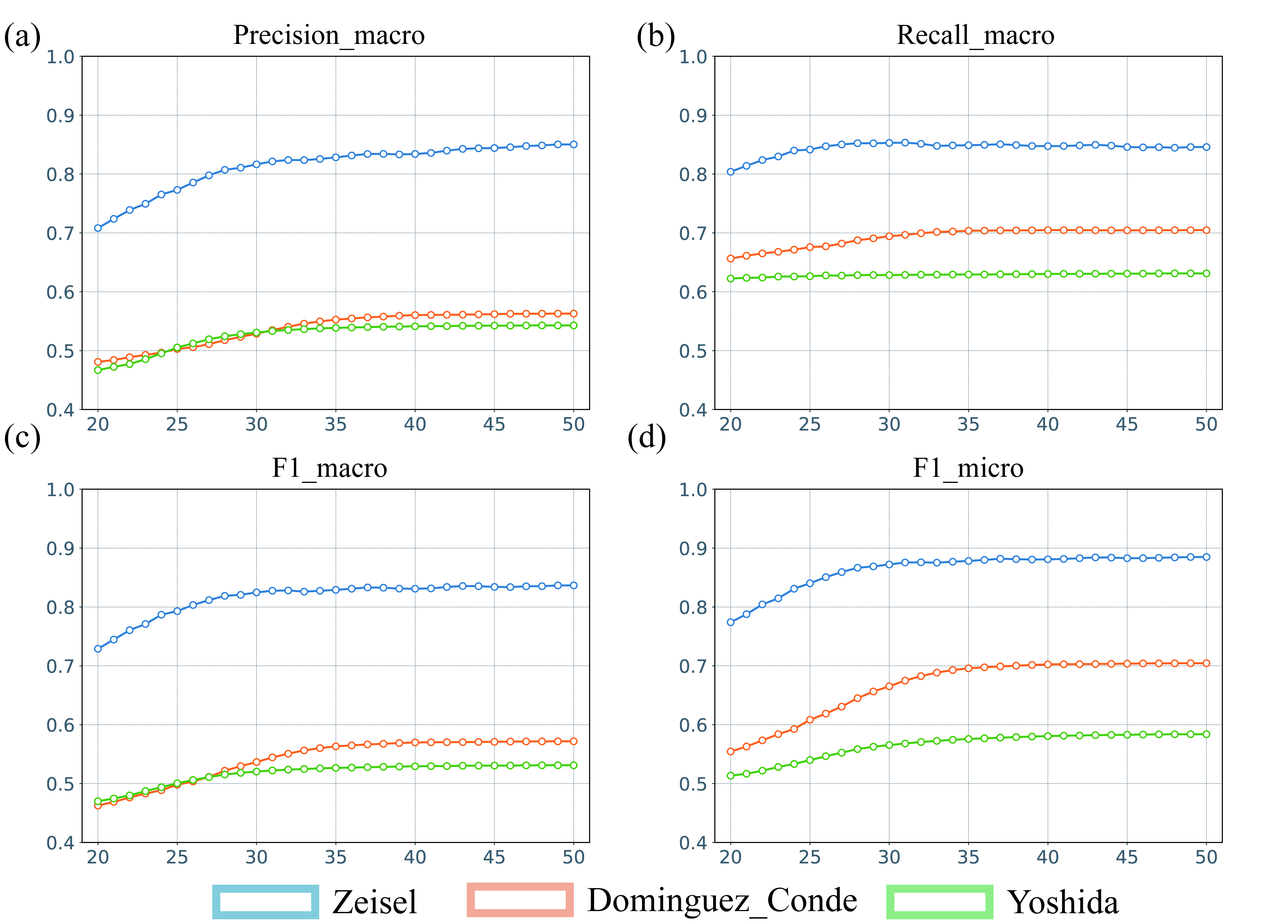

Supplement: btae569_Supplementary_Data [file btae569_supplementary_data.zip › Supplementary Fig. 1.tif]
